# Experimental Study on Mechanical Properties of Concrete at Super-Early Age

**DOI:** 10.3390/ma15217582

**Published:** 2022-10-28

**Authors:** Qiuwei Yang, Yun Sun, Xi Peng

**Affiliations:** 1 School of Civil and Transportation Engineering, Ningbo University of Technology, Ningbo 315211, China; 2 Engineering Research Center of Industrial Construction in Civil Engineering of Zhejiang, Ningbo University of Technology, Ningbo 315211, China

**Keywords:** concrete, super-early age, robust regression analysis, cohesion, friction angle

## Abstract

Few studies have reported the cohesion and friction angle of concrete at a super early age. However, these two mechanical parameters are necessary to study the influence of engineering vibration on super-early-age concrete. In view of this, the mechanical properties of the super-early age-concrete are investigated in this work by direct shear testing. Firstly, the shear strength of the super-early-age concrete is measured by the direct shear experiment under different normal pressures at different times. Secondly, the cohesion and friction angle of the super early age concrete are calculated according to the Mohr–Coulomb criterion of failure. To overcome the great discreteness and randomness in the measured data, a new robust regression analysis algorithm is presented to replace the traditional regression analysis method to obtain more reliable and reasonable mechanical parameters. According to the experimental and theoretical analysis results, it is found that the friction angles of the super early age concrete are located in the interval of [50°, 70°]. The cohesion of the concrete is about 78.7 kPa at the initial setting state and about 190.9 kPa at the final setting state, respectively. It has been shown that the cohesion of the concrete at a super-early age tends to increase rapidly with time. The method and test results of this work can be used as a reference for relevant engineering practice. Specifically, the proposed regression method can be extended to the data analysis of other mechanical parameters of concrete, as well as other brittle materials such as rock. The test results of early concrete cohesion and friction angle can be used to analyze the adverse effects of vibration on newly cast concrete members in pile driving and blasting engineering.

## 1. Introduction

In the construction projects of civil engineering, great attention is paid to the physical and mechanical properties of the concrete at an early age [1,2,3]. The mechanical properties of concrete after curing can be predicted by investigating the development law of the mechanical properties of concrete at an early age. On the flip side, the curing conditions of concrete can also be improved by the change rule of the mechanical properties of concrete at an early age. In some engineering practice, it is often necessary to discuss the impact of vibration on the mechanical properties of early-age concrete. To this end, much of the literature in recent years has reported the physical and mechanical properties of concrete at an early age, such as compressive strength, tensile strength, temperature, moisture, shrinkage, creep, impact resistance, etc.

To determine early-age strength, Swaddiwudhipong et al. [4] carried out the direct tension experiment to investigate the tensile strain capacity of the concrete with uniaxial tension at an early age. The experimental results indicated that the concrete tensile strain capacity is a relatively independent parameter. Yoshitake et al. [5] carried out the direct tension experiment to determine the tensile Young’s moduli of six concrete and mortar mixtures. It is found that the tensile modulus is about 1.0–1.3 times the compressive modulus in the first week of the material. Hasan and Kabir [6] developed a reasonable polynomial mathematical model to predict the compressive strength of concrete at 28 days, based on early results. Peng et al. [7] proposed an improved hyperbolic model to predict the compressive strength of the early-age concrete. Wolfs et al. [8] proposed a numerical model to analyze the mechanical properties of early 3D printing concrete. They found that in terms of the compressive and shear strength, Young’s modulus linearly increases with fresh concrete age. Wan et al. [9] investigated the early-age experimental characterization of the ultra-high-performance concretes. Based on the tested results, they proposed a computational model for the aging analysis of the ultra-high-performance concretes. Shen et al. [10] investigated the influence of internal curing on the early expansion of high-strength concrete. It was found that the early expansion increased with the increase in internal curing water. For the early-age temperature and moisture, Shen et al. [11] used the temperature stress test machine to study the effect of polypropylene plastic fiber length on the early age properties of the high-strength concrete. Azenha et al. [12] performed the experimental study to monitor concrete deformations during early ages after casting. It was found that the temperature sensitivity of the sensor to the curing instant of concrete and its signal during the pre-curing period is the key. Wu et al. [13] developed a finite element method based on ANSYS software to compute the early temperature stress field of concrete. The theoretical solution obtained by their method is very close to the experimental value. Zhang et al. [14] performed the experimental study to obtain the variation law of the moisture transfer coefficient for early-age concrete. They found that the moisture diffusion coefficient at the early age of concrete has a great relationship with the age. Zhang and Huang [15] proposed a general moisture distribution model for early-age concrete. Their model can well predict water distribution and its change over time. Hilaire et al. [16] employed a theoretical model to forecast the massive concrete cracking at the early age. Using the humidity and deformation sensors, Zhang et al. [17] investigated the change rule of internal relative humidity and global deformation for the early age concrete after casting. It was found that the global deformation behaves first as a plastic swelling, and then shrinking occurs in the initial few hours after casting.

For the early-age shrinkage and creep, Altoubat and Lange [18] found that the seal curing for the concrete samples cannot avoid the early-age shrinkage, but the wet curing can effectively release the shrinkage stresses. Di Luzio and Cusatis [19] proposed the solidification–microprestress–microplane (SMM) model to predict the mechanical properties of concrete at an early age and beyond. From the experimental data, it was found that their SMM model can describe the early shrinkage, creep and cracking of the concrete well. Soliman and Nehdi [20] studied the influences of drying conditions on the early shrinkage of high-strength concrete. They found that autogenous and drying shrinkage are dependent phenomena. Using fibre Bragg gratings, Wong et al. [21] found that the total shrinkage of the reactive powder concrete for seven days is 488 με, with the early age shrinkage contributing about 77% of this. Zhao et al. [22] studied the early creep of fly ash mass concrete using a thermal stress tester. They found that the ultra-high content fly ash concrete had only medium strength at the early stage, and had poor fresh and hardened engineering performance. Cortas et al. [23] studied the influence of aggregate water saturation on early shrinkage development and cracking risk of concrete. It is found that the development of early performance of concrete depends on the amount of water added in the mixing process. Liu et al. [24] found that multiaxial compression caused by early temperature rise in mass concrete can strengthen concrete and reduce tensile cracks. Farah et al. [25] conducted early bending creep tests on concrete beams and found that the Young’s modulus and residual strength of partially damaged beams decrease under creep load. Østergaard et al. [26] found that the concrete will show higher tensile creep strain if it is loaded at the age of less than or equal to one day. For the early-age impact resistance, Ansell and Silfwerbrand [27] summarized the experience in actual project construction and proposed the maximum vibration levels for the early age concrete. Dunham et al. [28] studied the influence of vibration on concrete strength during initial setting and final setting. Wojtowicz et al. [29,30] discussed the impact range and influence mechanism of vibration generated by pile driving on the newly cast reinforced concrete members.

However, few studies have reported the cohesion and friction angle of super-early-age concrete. In pile driving and blasting engineering, these two mechanical parameters are necessary parameters to study the influence of vibration on super-early-age concrete. Herein, the term super early age refers to the period between initial setting and final setting of the concrete. The cohesion and friction angle are important parameters to determine whether cracks occur in the super-early-age concrete under external vibration load such as blast, vehicle vibration, and so on. The research significance of this work mainly lies in two aspects. The first is to investigate the mechanical properties of the super-early-age concrete by direct shear testing. The obtained cohesion and friction angles of the early-age concrete can be used to evaluate the damage effect of vibration on fresh concrete members. The second is to propose a new regression algorithm to compute the cohesion and friction angle of the super-early-age concrete. This new regression approach can also be employed to analyze the other mechanical characteristics of the brittle materials. From the test and theoretical results, it is shown that the friction angle of the super-early-age concrete has little relationship with concrete age, but the cohesion is closely related to the age of concrete. Generally, the cohesion of concrete increases with the increase in concrete age. The framework of the study is presented as the following. Section 2 provides the experimental materials and process of the direct shear test for the concrete at a super-early age. Section 3 describes the main principles and formulas of the new regression algorithm. Finally, the conclusions of this study are summarized in Section 4.

## 2. Experimental Materials and Process

**Figure 1:**
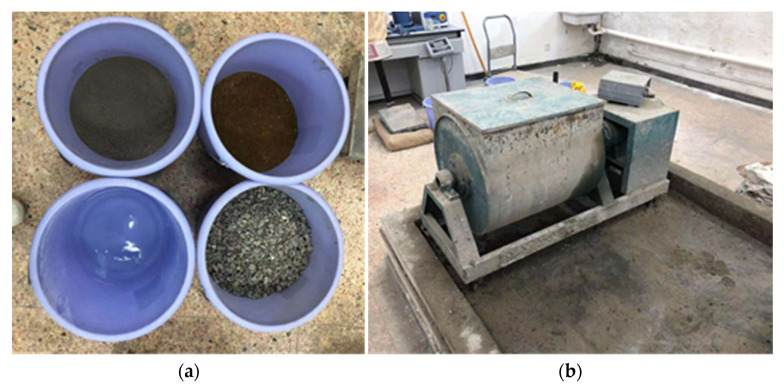
(**a**) Experimental materials; (**b**) Concrete mixer.

As stated before, there are few reports on the experiments of cohesion and friction angle of the concrete at a super-early age. However, these two parameters may be used to analyze the influence of vibration on the concrete at a super-early age. To this end, direct shear experiments are carried out in this work to obtain the cohesion and friction angle of the concrete at super-early age. This experiment is implemented by the method similar to the soil direct shear test. As seen in Figure 1a, the materials used in this experiment are cement, fine aggregate (natural yellow sand), water and coarse aggregate. Table 1 presents the concrete mix proportion in this experiment.

**Figure 2:**
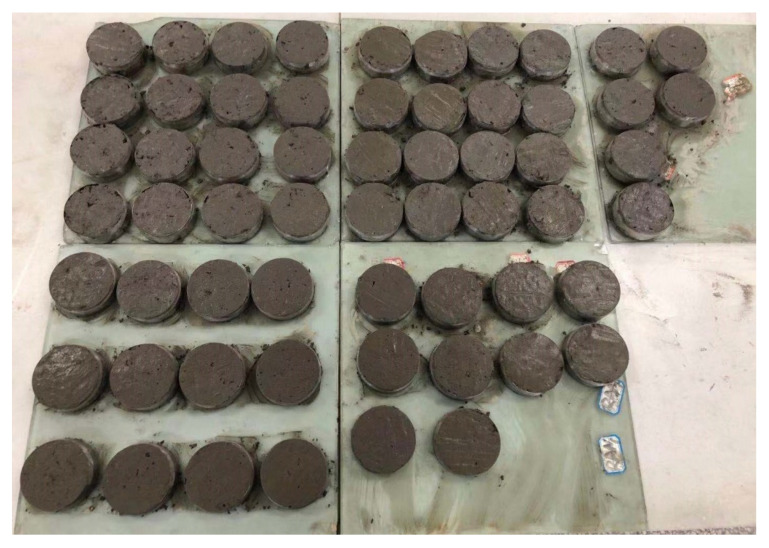
Round concrete specimens for direct shear test.

**Figure 3:**
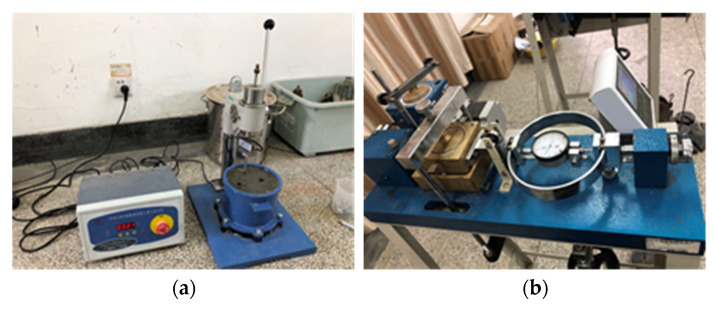
(**a**) Concrete penetration resistance instrument; (**b**) Direct shear instrument.

**Figure 4:**
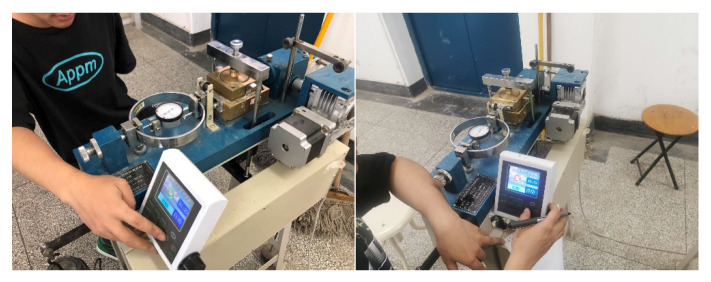
Direct shear test process.

**Figure 5:**
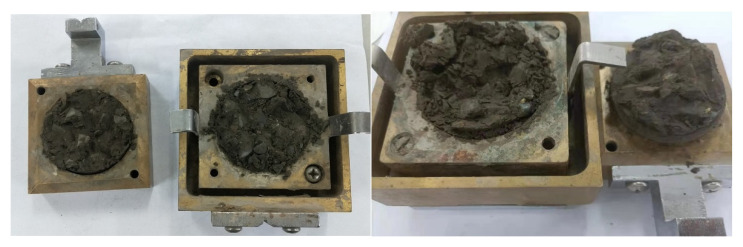
Direct shear failure modes of the samples.

At 8:50 a.m., the above materials were poured into the concrete mixer (shown in Figure 1b) to begin mixing. Subsequently, the round concrete specimens, as shown in Figure 2, were produced for the direct shear tests at different times. Figure 3 shows the two pieces of equipment used in the test: a concrete penetration resistance instrument and direct shear instrument. The concrete penetration resistance instrument is used to measure the penetration resistance of the concrete at different times. The setting state of concrete can be assessed by the measured values of the penetration resistance for the early-age concrete. The direct shear instrument is used to measure the shear strength of the concrete under different normal pressures at different times. Figure 4 presents the experiment process of the direct shear test. Figure 5 presents the common failure modes of the direct shear test for these samples.

Table 2 presents the tested values of the penetration resistance for the concrete at different times. Table 3 presents the tested values of the shear strength for the concrete under different normal pressures at different times.

The setting state of the concrete can be determined according to Table 2. In general, the penetration resistance value in the initial setting stage of concrete is located in the interval of [3.5, 6.9]. The penetration value of concrete at the final setting stage is generally in the interval of [19.6, 30.9]. Thus, one can see from Table 2 that the concrete was in the initial setting state at 12:11 a.m., and in the final setting state at 15:05 p.m. From Table 3, one can find that the shear strength increases with the increase in normal pressure for the concrete samples at each time. However, there is no obvious regularity in the shear strength data at different times. This reflects the great discreteness and randomness of mechanical properties of the concrete.

## 3. Regression Analysis Method

According to the Mohr–Coulomb criterion of failure, the relationship between shear strength τ and normal stress σ of the super-early-age concrete can be expressed as

(1) τ=c+σ⋅tan(φ)

where c and φ are the cohesion and friction angle of the concrete, respectively. Using Equation (1) and Table 3, the cohesion and friction angle of the super-early-age concrete can be calculated by the linear regression analysis. Traditionally, the linear regression analysis is based on the least-square solution. Letting $c=x_{0}$ and $\tan(\varphi )=x_{1}$, Equation (1) can be rewritten as the following linear equation

(2) $$\tau =\sigma \cdot x_{1}+x_{0}$$

For each time point, the regression coefficients $x_{0}$ and $x_{1}$ can be calculated by the least-square solution. Taking 12:11 a.m. as a case, the linear regression system for this time point can be obtained by substituting the data in Table 3 into Equation (2) as

(3) $$\left\{\tau \right\}=\Pi \cdot \left\{x\right\}$$

(4) $$\left\{\tau \right\}=\left\{\begin{array}{c} 203.6\\ 372.9\\ 481.1\\ 578.3 \end{array}\right\},$$

(5) $$\Pi =\left[\begin{array}{cc} 100 & 1\\ 200 & 1\\ 300 & 1\\ 400 & 1 \end{array}\right],$$

(6) $$\left\{x\right\}=\left\{\begin{array}{c} x_{1}\\ x_{0} \end{array}\right\}$$

Note that the values in Equations (4) and (5) are from Table 3, that and the corresponding units are kPa. In Equation (6), $x_{0}$ is the cohesion values with a unit of kPa, and $x_{1}$ is a pure number without unit. It should be pointed out that Equation (3) is the overdetermined system of the equation. From Equation (3), the regression coefficients $x_{0}$ and $x_{1}$ are often computed by the least-square estimation as.

(7) $${\left\{x\right\}}_{c}={\left(\Pi^{T}\Pi \right)}^{-1}\Pi^{T}\left\{\tau \right\}$$

in which the symbol “*T*” indicates the transposition process of a matrix. For the other time points, the regression coefficients $x_{0}$ and $x_{1}$ can also be computed by this regression approach based on the least-square estimation. All the calculated results of the regression coefficients $x_{0}$ and $x_{1}$ are shown in Table 4. Note that $x_{0}$ is the cohesion c. The friction angle φ is also given in Table 4, which is calculated by $\varphi =\arctan(x_{1})$.

From Table 4, it was found that the friction angles of the super-early-age concrete are located in the interval of [50°, 70°]. However, the cohesion values obtained by the traditional regression analysis in Table 4 are not very satisfactory. It is obvious that the calculated cohesions for 12:41 a.m., 14:00 p.m., 14:35 p.m., and 15:05 p.m. are wrong since, these values are abnormally small. The possible reason for this error is that the concrete mechanical properties may be very discrete and random in many cases. In view of this, the least-square regression algorithm cannot be successfully used to deal with those test parameters of concrete. Therefore, it is significant to develop new regression algorithms to tackle the discrete data. For this purpose, a regression analysis method based on modified weight coefficients is proposed to calculate the fitting parameters more accurately. The basic principle of the proposed method is to reduce the weight of the abnormal data according to the feedback of residual errors. At first, the residual error vector $\left\{v\right\}$ is derived from Equation (3) as

(8) $$\left\{v\right\}=\left|\Pi \cdot {\left\{x\right\}}_{c}-\left\{\tau \right\}\right|$$

The i-th element $v_{i}$ in $\left\{v\right\}$ is the absolute value of the residual error between the tested shear strength, with the analytical shear strength obtained by Equation (7). Assuming $m_{v}$ and $s_{v}$ are the mean and standard deviation of $\left\{v\right\}$, the relative deviation $r_{i}$ can be defined as

(9) $$r_{i}=\left|v_{i}-m_{v}\right|-s_{v}$$

From Equation (9), a diagonal matrix whose nonzero elements are weight coefficients can be defined as:

(10) $$W=\left[\begin{array}{cccc} w_{1} & 0 & 0 & 0\\ 0 & \ddots & 0 & 0\\ 0 & 0 & w_{i} & 0\\ 0 & 0 & 0 & \ddots \end{array}\right],$$

(11) $$w_{i}=\left\{\begin{array}{cc} e^{\frac{v_{\min}}{v_{i}}-1} & ,\;if\;r_{i}<0\\ 0 & ,\;if\;r_{i}\geq 0 \end{array}\right.$$

in which $v_{\min}$ is the minimum value of $\left\{v\right\}$. Using Equation (10), the solution of the unknowns $\left\{x\right\}$ can be computed by the new regression method as

(12) $${\left\{x\right\}}_{r}={\left(\Pi^{T}W^{2}\Pi \right)}^{-1}\Pi^{T}W^{2}\left\{\tau \right\}$$

Using Equation (12), the new solution of the regression coefficients $x_{0}$ and $x_{1}$ is listed in Table 5. From Table 5, it was found that the calculated friction angles are also located in the interval of [50°, 70°]. Comparing Table 5 with Table 4, one can see that the cohesion values obtained by the proposed regression method are more reasonable and reliable than those obtained by the traditional regression method. Using the proposed regression method, only the calculated cohesions of 12:41 a.m. and 14:00 p.m. are determined to be wrong, since these two values are abnormally small. Other cohesion values basically increase with the increase in time, and the distribution interval is more reasonable than that in Table 4. For example, the value 78.7 in Table 5 is more reasonable than the value 100.9 in Table 4 for 12.11 a.m. This is because the concrete is in the initial setting state at 12.11 a.m., and its cohesion should not be so large. For 16:20 p.m., the value 190.9 in Table 5 is more reasonable than the value 143.25 in Table 4. This is because the concrete is in the final setting state at 16:20 p.m., and its cohesion should be a lot larger.

**Figure 6:**
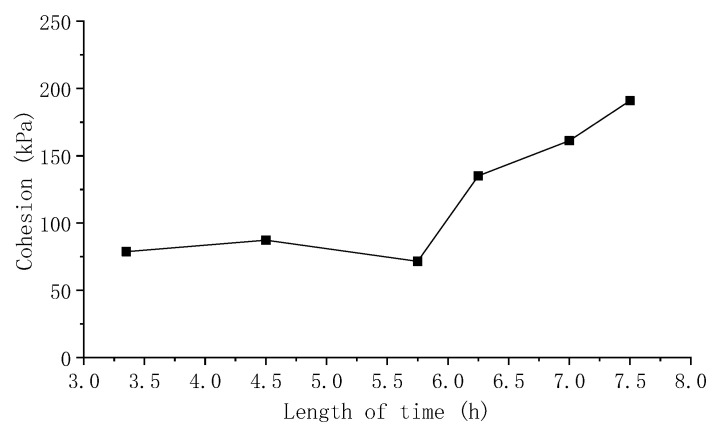
The changing trend of the valid cohesion–age data obtained by the proposed regression analysis.

From the above results, it was found that the friction angle of the concrete has little relationship with concrete age, but the cohesion is closely related to the age of concrete. Generally, the cohesion of concrete increases with the increase in age. From Table 5, the valid data for the relationship of concrete cohesion with age are listed in Table 6 and Figure 6. It is obvious that the cohesion of concrete at a super-early age tends to increase rapidly with time.

## 4. Conclusions

In this work, direct shear experiments are carried out to obtain the cohesion and friction angle of the concrete at a super-early age. A new robust regression analysis method is proposed to replace the traditional regression analysis method in order to analysis the measured data with great discreteness and randomness. From the test and numerical analysis, the following main conclusions can be obtained. (1) The existing regression algorithm using the least-square estimation is not suitable for computing some mechanical parameters of concrete, since the measured data are very discrete and random. (2) The proposed new regression algorithm can successfully overcome data discreteness and randomness, obtaining more reasonable and accurate calculation results. (3) It is found that the friction angles of the super-early age concrete are located in the interval of [50°, 70°]. (4) The cohesion of the concrete is about 78.7 kPa at the initial setting state and about 190.9 kPa at the final setting state, respectively. It is obvious that the cohesion of the concrete at a super-early age tends to increase rapidly with time. The theoretical and experimental results of this work can be used as references in related engineering practice. Specifically, the new regression method proposed in this paper can be extended to the data analysis of other mechanical parameters of concrete and can also be used in the data analysis of mechanical parameters of other brittle materials such as rock. The experimental results on the early cohesion and friction angles of concrete can be used to analyze the adverse effects of vibration on fresh concrete members in pile driving and blasting projects. It should be highlighted that this research is carried out only for one concrete mix. As the results are of practical importance, these tests can be continued in the future for different types of concrete and for different time parameters.

## Figures and Tables

**Table 1:** Concrete mix proportion.

Cement: Water: Sand: Coarse Aggregate = 1:0.43:1.22:2.27			
Cement	Water	Fine Aggregate	Coarse Aggregate
488.37 kg/m^3^	210 kg/m^3^	595.57 kg/m^3^	1106.06 kg/m^3^

**Table 2:** Tested values of the penetration resistance of the super-early-age concrete.

Time	12:11 a.m.	14:00 p.m.	15:05 p.m.	16:20 p.m.
The penetration resistance	3.87 MPa	6.24 MPa	21.8 MPa	31.5 MPa

**Table 3:** Tested values of the shear strength of the super-early-age concrete (kPa).

Time	The Shear Strength τ under Different Normal Pressure σ			
	σ = 100 kPa	σ = 200 kPa	σ = 300 kPa	σ = 400 kPa
12:11 a.m.	203.6	372.9	481.1	578.3
12:41 a.m.	192.8	374.7	482.8	756.7
13:20 p.m.	236.0	454.9	562.2	681.9
14:00 p.m.	151.4	414.4	351.4	924.3
14:35 p.m.	281.1	390.1	618.0	909.9
15:05 p.m.	324.3	320.7	500.0	891.9
15:50 p.m.	331.5	341.4	672.1	720.7
16:20 p.m.	264.9	434.2	454.9	677.5

**Table 4:** The regression coefficients obtained by the traditional regression analysis.

Time	12.11 a.m.	12:41 a.m.	13:20 p.m.	14:00 p.m.	14:35 p.m.	15:05 p.m.	15:50 p.m.	16:20 p.m.
$x_{1}$ $\left(\varphi =\arctan(x_{1})\right)$	1.2323 (50.94°)	1.7998 (60.94°)	1.4450 (55.32°)	2.2557 (66.09°)	2.1143 (64.69°)	1.8821 (62.02°)	1.4983 (56.28°)	1.2585 (51.53°)
$x_{0}$ ($c=x_{0}$, kPa)	100.9	1.8	122.5	−103.55	21.2	38.7	141.85	143.25

**Table 5:** The regression coefficients obtained by the proposed regression analysis.

Time	12.11 a.m.	12:41 a.m.	13:20 p.m.	14:00 p.m.	14:35 p.m.	15:05 p.m.	15:50 p.m.	16:20 p.m.
$x_{1}$ ($\varphi =\arctan(x_{1})$)	1.2490 (51.32°)	1.8738 (61.91°)	1.4863 (56.07°)	2.5820 (68.82°)	2.096 (64.49°)	1.892 (62.14°)	1.7030 (59.58°)	1.2165 (50.58°)
$x_{0}$ ($c=x_{0}$, kPa)	78.7	3.603	87.3667	−105.7337	71.5	135.1	161.2	190.9

**Table 6:** The valid cohesion–age data obtained by the proposed regression analysis.

Length of Time	3.35 h	4.5 h	5.75 h	6.25 h	7 h	7.5 h
Cohesion c (kPa)	78.7	87.3667	71.5	135.1	161.2	190.9

## Data Availability

The data generated and/or analyzed during the current study are not publicly available for legal/ethical reasons but are available from the corresponding author on reasonable request.
